# Niche selection in bacterioplankton: A study of taxonomic composition and single‐cell characteristics in an acidic reservoir

**DOI:** 10.1111/1758-2229.13255

**Published:** 2024-06-28

**Authors:** Sara Soria‐Píriz, Alfonso Corzo, Juan Luís Jiménez‐Arias, Juan M. González, Sokratis Papaspyrou

**Affiliations:** ^1^ Departamento de Biología, Facultad de Ciencias del Mar y Ambientales Universidad de Cádiz Puerto Real, Cádiz Spain; ^2^ Institute of Natural Resources and Agrobiology Spanish National Research Council, IRNAS‐CSIC Sevilla Spain; ^3^ Instituto Universitario de Investigacion Marina (INMAR), Campus Universitario de Puerto Real Universidad de Cádiz Puerto Real, Cádiz Spain

## Abstract

Niche selection and microbial dispersal are key factors that shape microbial communities. However, their relative significance varies across different environments and spatiotemporal scales. While most studies focus on the impact of these forces on community composition, few consider other structural levels such as the physiological stage of the microbial community and single‐cell characteristics. To understand the relative influence of microbial dispersal and niche selection on various community structural levels, we concurrently examined the taxonomic composition, abundance and single‐cell characteristics of bacterioplankton in an acidic reservoir (El Sancho, Spain) during stratification and mixing periods. A cluster analysis based on environmental variables identified five niches during stratification and one during mixing. Canonical correspondence analysis (CCA) revealed that communities within each niche differed in both, taxonomic and single‐cell characteristics. The environmental variables that explained the variation in class‐based ordination differed from those explaining the ordination based on single‐cell characteristics. However, a Procrustes analysis indicated a high correlation between the CCA ordinations based on both structural levels, suggesting simultaneous changes in the microbial community at multiple structural levels. Our findings underscore the dominant role of environmental selection in occupying different microbial niches, given that microbial dispersal was not restricted.

## Graphical abstract

The discrimination of six microbial potential niches in the water column of an acid mine drainage impacted reservoir in space and time, based on a set of 10 environmental variables, occurred simultaneously at the level of taxonomic composition, and at the level of abundance and single cell characteristics of the bacterioplankton communities.

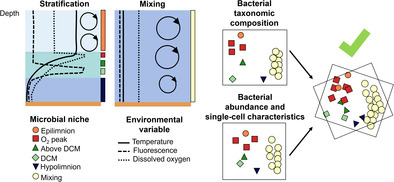

## INTRODUCTION

Bacterioplankton plays a crucial role in aquatic ecosystems, being a key player in biogeochemical cycling and channelling matter and energy to higher trophic levels (Azam, 1998; Cotner & Biddanda, 2002). Bacterioplankton presents a scarcely studied multilevel community structure that can affect qualitative and quantitatively those ecological functions. This multilevel community structure includes, in addition to its taxonomic composition, the range of different types of metabolisms, physiological stages and single‐cell characteristics, such as cell size (Comte & del Giorgio, 2009; del Giorgio & Gasol, 2008). In turn, different biotic and abiotic ecological drivers, for example, temperature, nutrients, grazing and dispersal rates, which change at different spatiotemporal scales, affect these features at various levels of biological organization (Comte & del Giorgio, 2009; del Giorgio & Bouvier, 2002; del Giorgio & Gasol, 2008; Gasol et al., 1999). Changes in the bacterioplankton community in response to environmental conditions have been observed so far at different structural levels at large spatial scales, for example, at the basin scale, along lengthy downstream systems and across latitudinal gradients (Comte & del Giorgio, 2009; Kuang et al., 2013; Niño‐García et al., 2016a, 2016b; Schiaffino et al., 2013). However, the simultaneous effect on both bacterioplankton taxonomic composition, using new‐generation sequencing approaches, and single‐cell characteristics have not been studied along environmental gradients at smaller spatiotemporal scales.

The scale as a factor in terms of space and time is relevant because it can affect microbial dispersal rates and therefore the degree of similarity between different niche‐associated microbial communities at different structural levels. The microbiota of any given environment (or niche) at any time unit is the result of dispersal rates and environmental selection (Niño‐García et al., 2016a; Pedrós‐Alió, 2006; Stegen et al., 2012) and depending on the dimension of the working spatiotemporal scale, the relative importance of both factors, that is, dispersal rates and environmental selection is different. On one hand, at large spatiotemporal scales, the distinction of the relative weight of both factors in shaping the observed microbial community is difficult because both dispersal (connectivity) and environmental selection can be highly variable and site‐specific. On the other hand, at lower spatial scales, (cm to tens of m), microbial dispersal rates and therefore connectivity between potentially different pelagic microbial niches, are likely to be high due to the typically large vertical turbulent diffusion coefficient observed in the water column (see, [Salas De León et al., 2016] and references therein), which will nullify or at least blur the differences in the microbial community, from taxonomic to single‐cell characteristics. Therefore, differences in the multilevel structure of the microbial community can only be observed if niche selection is a relevant factor. In addition, in such conditions, we can confidently ascribe any observed change in the microbial community to differences in the characteristics of their niches.

Lakes and reservoirs are appropriate systems to examine the dynamics of the bacterioplankton community structure in response to changes in environmental drivers and the resulting habitat heterogeneity in space and time within the same water body (Shade et al., 2008; Shade et al., 2012). The multilevel structure of the microbial community from taxonomy to single‐cell characteristics must adapt to cope with the striking changes in a wide set of abiotic and biotic environmental conditions triggered by the alternation between stratification and mixing periods (Wetzel, 2001). These changes occur with depth in the water column at scales of meters and in weeks to months in the time dimension. Here, we examine the relationship between the potential changes in the multilevel community structure of bacterioplankton and the environmental changes occurring during the stratification and mixing periods in El Sancho reservoir (SW Spain), an extreme acid environment (pH: 3.5–4.5) due to chronic pollution by acid mine drainage (AMD) (Torres et al., 2013). Acid mine drainage (AMD) impacted systems, in particular, have been long recognized as model systems for the quantitative analysis of microbial ecology and community function due to their relative biological simplicity (Huang et al., 2016) as a result of the extreme conditions (low pH and high concentration of toxic heavy metals), which affect the abundance and diversity of microbial communities (Nixdorf et al., 1998; Williamson et al., 2008; Zhang et al., 2019). In El Sancho, the annual thermal cycle is similar to that of other holomictic freshwater lakes as previous work has shown (Soria‐Píriz et al., 2019; Torres et al., 2013) and important changes in the microbial community during its annual cycle are expected as shown in neutral freshwater lakes (Morrison et al., 2017; Shade et al., 2012). However, the low pH and high metal concentrations of El Sancho make the study of its microbiota very interesting due to the scarce information available on the microbial communities of similar environments (Percent et al., 2008; Sánchez España, 2008).

In the present study, we aimed to (1) identify different microbial niches in the water column during the stratification and mixing periods of the annual cycle; (2) determine whether niche environmental differences drive the bacterioplankton taxonomic composition and single‐cell characteristics in the presence of high microbial dispersal rate; (3) assess the potential links between these two levels of the community structure in space and time; and (4) identify environmental factors that can explain this variability.

## EXPERIMENTAL PROCEDURES

### Study site and sampling

El Sancho (Huelva, Spain) is a mid‐size holomictic reservoir (58 hm^3^) located within the Iberian Pyrite Belt, one of the world's largest deposits of massive sulphide with a long mining history. El Sancho is heavily polluted by AMD due to the inflow of its main tributary (River Meca). El Sancho was initially neutral but is undergoing an acidification process, reaching at present a pH of 3.5–4.5 (Torres et al., 2013). During stratification, the hypolimnion becomes hypoxic and a deep chlorophyll maximum (DCM) develops in the metalimnion (Soria‐Píriz et al., 2019). Field measurements and water samplings were carried out at several depths at a single site (37°27′49″N, 6°59′3″W) located at the deepest part of the reservoir (~35 m depth) during stratification (September–October 2013) and mixing (January–February 2014) periods (Table 1).

**TABLE 1:** Dates and selected depths during stratification (summer) and mixing (winter) samplings in El Sancho reservoir.

Season	Date	Year	ID	Depths (m)
Stratification	12th September	2013	S1	0, 5, 10, 16, 20, 24, 28
Stratification	18th September	2013	S2	0, 5, 10, 16, 20, 23, 33
Stratification	25th September	2013	S3	0, 5, 10, 16, 20, 22, 33
Stratification	8th October^a^	2013	S4	0, 5, 10, 15, 20, 22, 33
Mixing	8th January^a^	2014	M1	0, 5, 10, 15, 20, 26, 32
Mixing	25th January^a^	2014	M2	0, 5, 10, 15, 20, 26, 32
Mixing	4th February^a^	2014	M3	0, 5, 10, 15, 20, 26, 32

^a^ Available DNA extraction data.

### Environmental variables

Water column vertical profiles of temperature, pH and fluorescence were obtained using a multiparameter probe (Hydrolab MS5). Temperature vertical profiles were used to calculate density vertical profiles, from then the Brunt‐Väisälä frequency and finally the vertical turbulent diffusion coefficient (*K*_d_, m^2^s^−1^) (Soria‐Píriz et al., 2019). High‐resolution dissolved oxygen (O_2_) profiles were determined with an O_2_ microsensor (MP4 Miniprofiler, UNISENSE).

Water samples were collected using a 4.2 L Van Dorn acrylic bottle (1140‐G42, Wildlife Supply Company) and properly stored for each subsequent analysis later in the laboratory (Soria‐Píriz et al., 2019). Dissolved O_2_ in water samples was analysed by the Winkler method spectrophotometrically following (Labasque et al., 2004). Total inorganic dissolved carbon (CO_2_) was analysed on an InfraRed Gas Analyser (Qubit systems, S151CO2 analyser) following the FIA setup of Hall and Aller (1992). Ammonium (NH_4_^+^) and nitrate plus nitrite (NO_x_) were analysed spectrophotometrically from GF/F filtered samples according to Bower and Holm‐Hansen (1980) and García‐Robledo et al. (2014), respectively. Chlorophyll a (Chl *a*) was collected on GF/F filters, extracted at 4°C for 12 h with 4 mL of acetone 90%, tubes centrifuged (2200 × g, 5 min), the absorbance read spectrophotometrically and concentrations determined according to Ritchie (2008). Particulate organic carbon (POC) collected on pre‐combusted (450°C, 4 h) GF/F filters was determined on a FlashEA1112 (Thermo Finnigan) elemental analyser (University of A Coruña, Central Services). Dissolved organic carbon (DOC) content was determined in 0.2 μM nylon filtered acidified (1 mL of phosphoric acid 1:3) water samples on a Shimadzu TOC‐5050 analyser (ICMAN‐CSIC, Central Services). Dissolved metals, that is, potassium (K), magnesium (Mg), calcium (Ca), aluminium (Al), soluble ferrous iron (Fe^2+^, abbreviated as Fe), Zinc (Zn), copper (Cu), Manganese (Mn), Sodium (Na) and Silica (Si), were determined in unfiltered acidified (0.5 mL of HCl 2 M, 0.2 M final concentration) water samples on an ICP‐OES analyser (CCiTUB, Universitat de Barcelona). Further methodological details about sample collection, storage and analyses can be found in Soria‐Píriz et al. (2019).

### DNA sequences

To examine the taxonomic composition of the bacterial community, water from each depth—from only one sampling (*n* = 7, unfortunately, two samplings were lost during long‐term storage) during stratification (summer) and from three samplings (*n* = 21) during mixing (winter)—was filtered through a Sterivex filter (0.22 μm, Millipore®) until the filter was saturated and then fixed with RNALater® (Qiagen). DNA extractions were performed with the PowerWater Sterivex DNA Isolation kit (MoBio Laboratories, Inc., Carlsbad, CA) with an additional initial wash in an acidic buffer (pH 1.8; 0.1 M KCl, 0.04 M HCl) followed by neutralization in TE buffer (10 mM Tris–HCl, 1 mM EDTA, pH 8.0) (Bond et al., 2000). Amplicon‐based libraries for the 16S rRNA gene were pyrosequenced on a FLX Titanium Genome Sequencer (Roche Applied Sciences, 454 Life Sciences, Branford, CT, USA) using universal primers 341F (5′‐CCTAYGGGRBGCASCAG‐3′) and 806R (5′‐GGACTACNNGGGTATCTAAT‐3′) (Yu et al., 2005). Pyrosequencing data generated from the 454‐sequencing runs were processed using QIIME (Caporaso et al., 2010). Briefly, chimera sequences were detected and removed using the UCHIME algorithm (Edgar et al., 2011). Quality sequences were binned into phylotypes (97% similarity) using UCLUST (Edgar, 2010) by the de novo procedure (Caporaso et al., 2010). Representative sequences were then aligned using PyNAST (Caporaso et al., 2010) with a phylogenetic tree constructed using the maximum likelihood algorithm implemented in FastTree (Price et al., 2010). Singleton OTUs were filtered out, and taxonomy was assigned to the OTUs using the RDP classifier (Wang et al., 2007). Sequence data have been deposited in the NCBI database under the accession number PRJNA720891. OTUs that were taxonomically annotated as eukaryotic ‘Chloroplast’ were excluded from the subsequent community analysis. Only phyla or class ≥1% of their relative abundance were considered individually if the average of the relative abundance across all the depths and samplings was ≥1%, while phyla or class with relative abundance <1% were pooled in the ‘Others’ category. Both the most (≥1%) and less (<1%) abundant phyla or classes were taking account in the analysis.

### Flow cytometry

Water samples were fixed in cryotubes (4.5 mL) using glutaraldehyde (1% final concentration) and frozen at −80°C until analysis. Samples were left to thaw at room temperature, and 1 mL aliquots were immediately stained with 10 μL of SYBR® Green‐I nucleic acid gel stain (Molecular Probes #S7563) (2.5 μM final concentration), and incubated for 10 min at room temperature in darkness (Corzo et al., 2005; Lebaron et al., 2001). A known amount of autofluorescent beads (1.1 μm diameter, Ex/Em: 430/465 nm, FluoSpheres® Molecular Probes Inc.™) were added to each sample as an internal standard to eliminate potential differences between flow cytometers and different runs in the same machine. Reference beads and stained bacterial cells were excited at 488 nm and detected according to their 90° light scatter (SSC) and green fluorescence (FL). Samples from stratification and mixing were analysed on Dako CyAnTM ADP (Beckman Coulter™) and CytoFLEX (Beckman Coulter™) flow cytometers respectively. All samples were analysed at medium flow rate and were run until at least 30,000 counts were recorded. Data were treated with FCS Express De Novo™ Software.

Two heterotrophic bacterial subpopulations were differentiated in all samples based on their signature in the FL versus SSC cytograms: the so‐called high nucleic acid (HNA) and low nucleic acid (LNA) contents subpopulations (Corzo et al., 2005; Gasol & del Giorgio, 2000; Lebaron et al., 1998). In addition to quantifying the changes in abundance of the subpopulations, we analysed the changes in two cellular characteristics for every HNA and LNA classified cell: SSC, as a proxy of cell size, granularity and complexity, and FL intensity, as a proxy of nucleic acid cellular content (Gasol et al., 1999; Lebaron et al., 2002; Morán et al., 2007). The FL‐to‐SSC ratio by cell (FL/SSC) is used as a proxy of cellular nucleic acid content normalized to cell size (Gasol & del Giorgio, 2000). Mean cell values of SSC and FL for the HNA and LNA subpopulations were always referred to as the corresponding mean values of the autofluorescent beads (Gasol & del Giorgio, 2000).

### Statistical analysis

Environmental variables (i.e., Temperature, O_2_, pH, CO_2_, NH_4_^+^, NO_x_, Chl *a*, POC and DOC), dissolved metals (K, Mg, Ca, Al, Fe, Zn, Cu, Mn, Na and Si), total bacterial abundance, HNA and LNA abundances, FL, SSC and FL/SSC of HNA and LNA fractions were standardized (0–1) to remove differences in scales prior to multivariate statistical analyses (Legendre & Legendre, 2012).

A principal component analysis (PCA), based on the Euclidean dissimilarity matrix of the abiotic variables including dissolved metals, was performed using the prcomp function (R stats package version 3.6.2). Environment variables that contributed >10% in both PCA1 and PCA2 components to the ordinations of the depths were selected to identify potential niches. These variables were T, pH, O_2_, CO_2_, NH_4_^+^, DOC, NO_x_, and Ca (Figure S1). A similarity profile analysis (simprof), based on the Euclidean dissimilarity matrix of this selection, also including Chl *a* and POC due to their sharp vertical distribution during stratification (Soria‐Píriz et al., 2019), was performed to identify contrasting environmental conditions, that is, potential niches which could harbour different bacterial communities and cellular characteristics, using the hclust() function with 9999 permutations, the ‘average’ method and with *α* = 0.05 (R package ‘clustsig’ version 1.1). The results were visualized in a non‐metric multidimensional scaling plot (nMDS) (R package vegan version 2.5‐6) (Oksanen et al., 2019).

Non‐metric multidimensional scaling (nMDS) ordination analysis of Bray Curtis distance of the most abundant phylum, class, genus and OTUs were tested to test for possible differences in sample ordination dependent on the taxonomic level used (metaMDS() function, R package vegan version 2.5‐4) (Oksanen et al., 2019). The congruence among the nMDSs was tested using the procrustes() function (R package vegan version 2.5‐6) (Oksanen et al., 2019) (Figure S2). This method determines the best superimposition that maximizes the fit through scales and rotations processes by matching corresponding points (depths in this study) from the four multivariate data sets. The significance of the Procrustes statistics was evaluated using the protest() function with 9999 permutations (R package vegan version 2.5–6) (Oksanen et al., 2019).

Two different canonical correspondence analyses (CCA) (R package Vegan version 2.5–6) (Oksanen et al., 2019) were used to explain the variation in taxonomic composition at the class level and single‐cell characteristics through the water column and between seasons, in relation to the environmental variables. In the first, the most abundant classes were used as ‘species’, whereas in the second we used abundance and single‐cell characteristics (i.e., FL, SSC) of both HNA and LNA and % HNA. Environmental variables were used as constraining variables in both cases. Collinearity of environmental variables was checked (ggpairs() function, R package GGally: Extension to ‘ggplot2’ version 1.4.0) (Schloerke et al., 2018) and variables with correlation >0.8 were eliminated. Temperature highly correlated with NO_x_; NH_4_^+^ with CO_2_; Chl *a* with POC; Fe with K; and Ca with Mg, Cu, Al, Zn, Na and Si, thus, only temperature, NH_4_^+^, Chl *a*, Fe and Ca were kept. A forward‐selection procedure using the Akaike information criterion with 9999 permutations was used to choose the environmental variables best explaining the variations in each CCA (*p* < 0.05) (R package vegan version 2.5‐6) (Borcard et al., 1992; Oksanen et al., 2019). The congruence between both CCAs was tested using the procrustes() function (R package vegan version 2.5‐6) (Oksanen et al., 2019).

To assess the significant variability of the total bacterial abundance and the %, FL and SSC of HNA and LNA cells through the water column and between seasons, we used one‐way ANOVA and Tukey–Kramer means post‐hoc comparison tests (*α* = 0.05).

## RESULTS AND DISCUSSION

### Diversity of microbial niches in the water column in space and time

**FIGURE 1:**
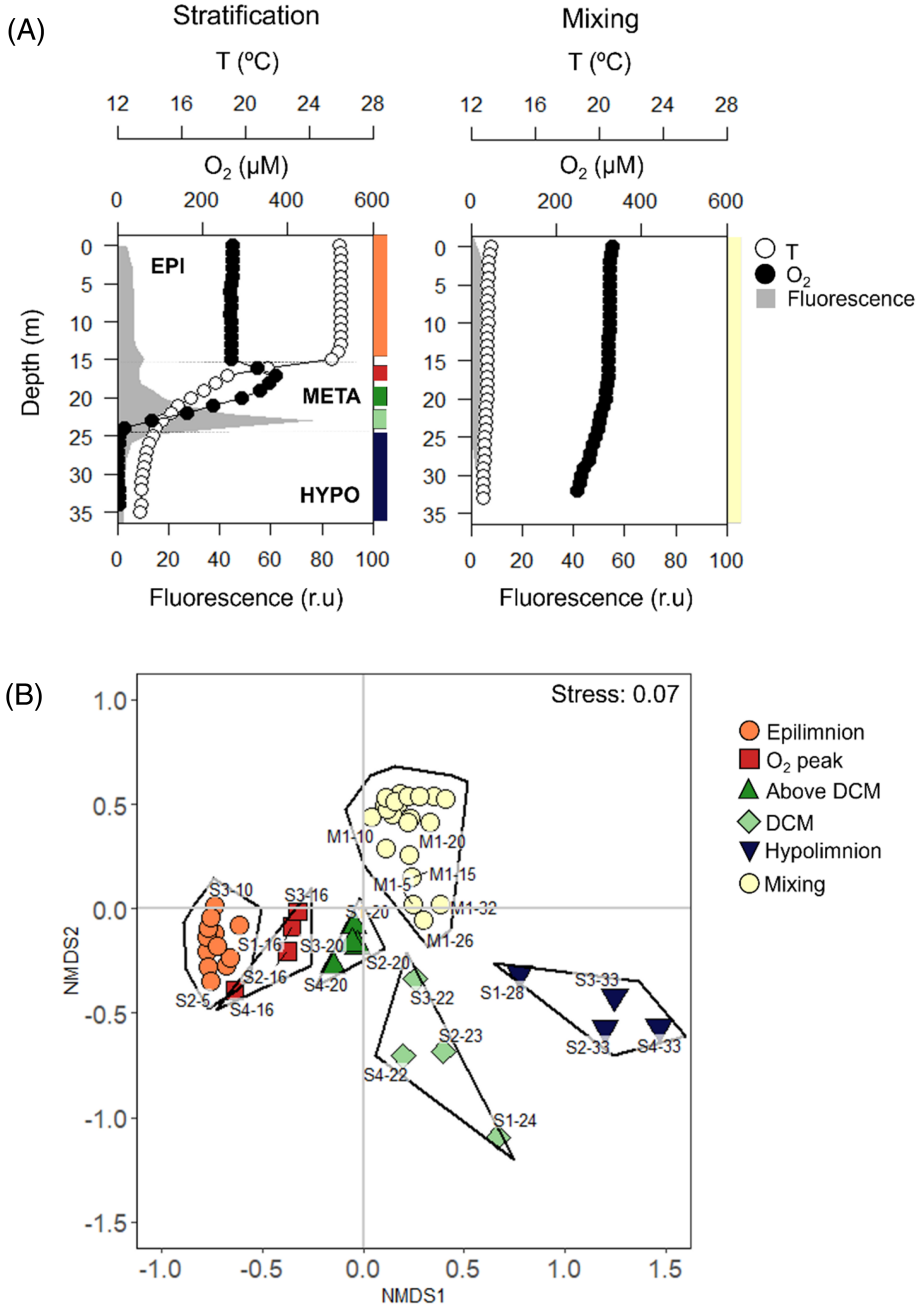
(A) Depth profiles showing the vertical distribution of temperature (*T*), dissolved oxygen (O_2_) and fluorescence in El Sancho reservoir during stratification and mixing. Epilimnion (EPI), metalimnion (META) and hypolimnion (HYPO) layers are distinguished in the left plot. (B) Non‐metric multidimensional scaling (nMDS) plot of studied samples of all environmental variables based on Euclidean distance. Based on SIMPROF similarity profile analysis (*α* = 0.05), five clusters during stratification (Eplimnion, O_2_ peak, Above DCM, DCM and hypolimnion) and one during mixing (mixing) were identified as possible potential niches which could harbour different bacterial communities in space and time. The positions of the niches in the water column (panel A) are plotted with the same colours as they appear in the nMDS plot (panel B). S1, S2, S3 and S4 are the four samplings during the stratification season and M1, M2 and M3 are the three samplings during the mixing season. The numbers beside are the different depths.

The water column in El Sancho included a wide range of environmental conditions from the highly illuminated, warm, and oxic epilimnion to the low light conditions prevailing above the DCM, and the dark, cold and hypoxic conditions prevailing in the hypolimnion (Figure 1A, B, Table S1). The environmental differences included different degrees of water column stability due to the thermal structure of the water column during stratification and mixing as estimated by vertical turbulent diffusion coefficient (*K*_d_). *K*_d_ changed from 1.9 × 10^−4^ m^2^ s^−1^, for a homogeneous water column during mixing, to 5.2 × 10^−7^ m^2^ s^−1^, three orders of magnitude lower in the metalimnion during stratification. Intermediate values were measured in the epilimnion and hypolimnion, 7.3 × 10^−5^ and 8.6 × 10^−6^ m^2^ s^−1^, respectively (Table S1, Soria‐Píriz et al., 2019). *K*_d_ can be used as a proxy of the vertical microbial dispersal rate in the water column and therefore, the mean distance a non‐motile bacteria moves from its original position (L) for a given time (*t*) calculated from the Einstein‐Smoluchowski equation ($L=\sqrt{2K_{d}t}$). The calculation of *L* supported our hypothesis, suggesting that turbulent diffusion facilitates high vertical bacterial dispersal rates, transporting bacterial cells from their original position a mean distance of 3.5 m in the epilimnion, 1.2 m in the hypolimnion, 0.3 m in the metalimnion and 5.7 m during the mixing period in just 1 day. This indicates that in water layers with potentially lower vertical bacterial dispersal rates, the development of different potential microbial niches and therefore different communities are favoured.

During stratification, cluster analysis identified four potential niches with different environmental conditions. These niches were associated with the epilimnion (0–10 m depth), the metalimnetic O_2_ maximum (‘O_2_ peak’, 16 –20 m depth), the DCM (22–24 m depth), where the maxima of fluorescence, Chl *a* and POC occurred, and the hypolimnion (28–33 m depth) (Table S1). In addition, we decided to distinguish a fifth potential niche at 20 m depth and separate it from the ‘O_2_ peak’ (16 m depth). This niche corresponded to the upper layer of the DCM (‘above DCM’), where the maximum net production was found (Soria‐Píriz et al., 2019) (Figure 1B, Table S1). During mixing, the whole water column was identified as a single microbial niche (Mixing, 0–32 m depth), since changes in the biogeochemical variables were minimal (Figure 1B, Table S1).

### Taxonomic composition of bacterioplankton

A total of 1,922,474 sequences were generated, averaging 13,000 sequences per sample, comprising a total of 20,186 operational taxonomic units (OTUs). Diversity indices (Shannon, Simpson, Chao1) based on OTUs relative abundance increased with depth during stratification whereas they were more homogeneous in mixing (Figure S3). The increase of bacterial abundance and diversity (OTUs, classes and phyla) with depth (Figures 3 and S3–S5) has been recorded in other lakes as well (Nelson, 2009; Peura et al., 2012). This pattern is likely explained by the higher availability of potential substrates like DOC and particulate organic carbon and nitrogen. These organic substrates originate from the decomposition of phytoplanktonic detritus, evidenced by the increase in NH_4_^+^, CO_2_ and DOC concentrations with depth (Table S1) (Soria‐Píriz et al., 2019). In addition, the decrease in O_2_ availability with depth can favour both the increase in microbial diversity and the change in taxonomic composition as well, since the higher chemical diversity of inorganic and organic substrates and potential electron acceptors favours taxonomic diversity (Barberán & Casamayor, 2011; Stevens & Ulloa, 2008).

**FIGURE 2:**
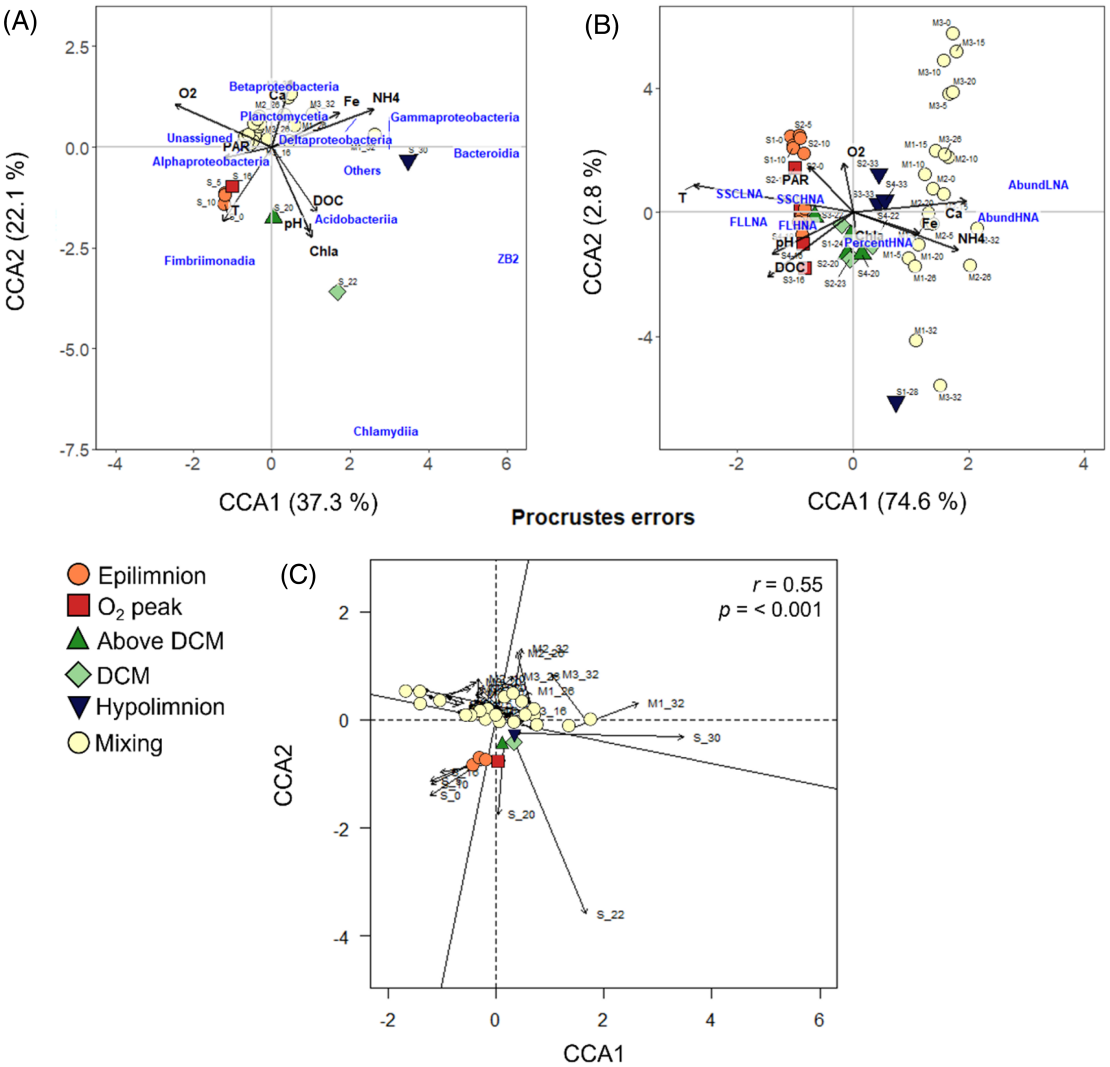
(A) Canonical correspondence analysis (CCA) ordination of microbial niches distinguished in El Sancho (Figure 1C, Table S1) based on the relative abundance (%) of the most abundant bacterial classes. Symbols represent sampling points. Correlations between environmental variables and the microbial niche ordination are represented by the black arrows. Data are from one sampling during stratification (October 2013) (*n* = 7) and three samplings during mixing (January–February 2014) (*n* = 21). (B) CCA ordination of the microbial niches based on the abundances and cytometric characteristics (FL and SSC) of HNA and LNA subpopulations and % of HNA (bold letters). Symbols represent sampling points. Correlations between environmental variables and the microbial niche ordination are represented by arrows. Data are from four samples during stratification (September–October 2013) (*n* = 28) and three samplings during mixing (January–February 2014) (*n* = 21). The abundances and the single cell cytometric characteristics (FL and SSC) and environmental data were scaled (0–1). (C) Procrustes superimposition plot comparing both CCAs. Notice that to compare both CCAs with Procrustes, it was necessary to do another CCA for the abundances and cytometric characteristics (FL and SSC) of HNA and LNA with the average per depth of the four sampling during stratification (from *n* = 28 to *n* = 7) since there was only one sampling during stratification (October 2013, *n* = 7) for the most abundant bacterial classes. Lines represent the Procrustes residual from both canonical correspondence analysis ordinations in each microbial niche. Correlation (*r*) and significance values (*p*) were calculated using the protest function (Vegan R package).

Bacterial communities can be characterized at different taxonomic levels depending on the ecological question of interest. Since no previous information existed on El Sancho microbiota nor in any comparable system, a top‐down approach beginning with higher taxa (phylum and class) seems more suitable. In addition, we checked that the ordination of the samples at successive taxonomic levels, that is, phyla, classes, genus and OTUs, produced the same ordination pattern between the distinct niches (Figure S2). Thus, ordination analysis was based on the most abundant bacterial classes revealing their clear separation in the different biogeochemical niches (Figure 2A). Variation at class level was explained significantly by axes CCA1 (37.2%, *F*_1,18_ = 27.6, *p* = 0.001), and CCA2 (22.1%, *F*_1,18_ = 16.4, *p* = 0.001) (Figure 2A). NH_4_^+^, O_2_, Chl *a*, DOC, temperature and Fe, the variables selected by the Forward‐selection procedure, explained together 58.6% of the variation in the bacterial taxonomic composition‐based ordination at the class level.

**FIGURE 3:**
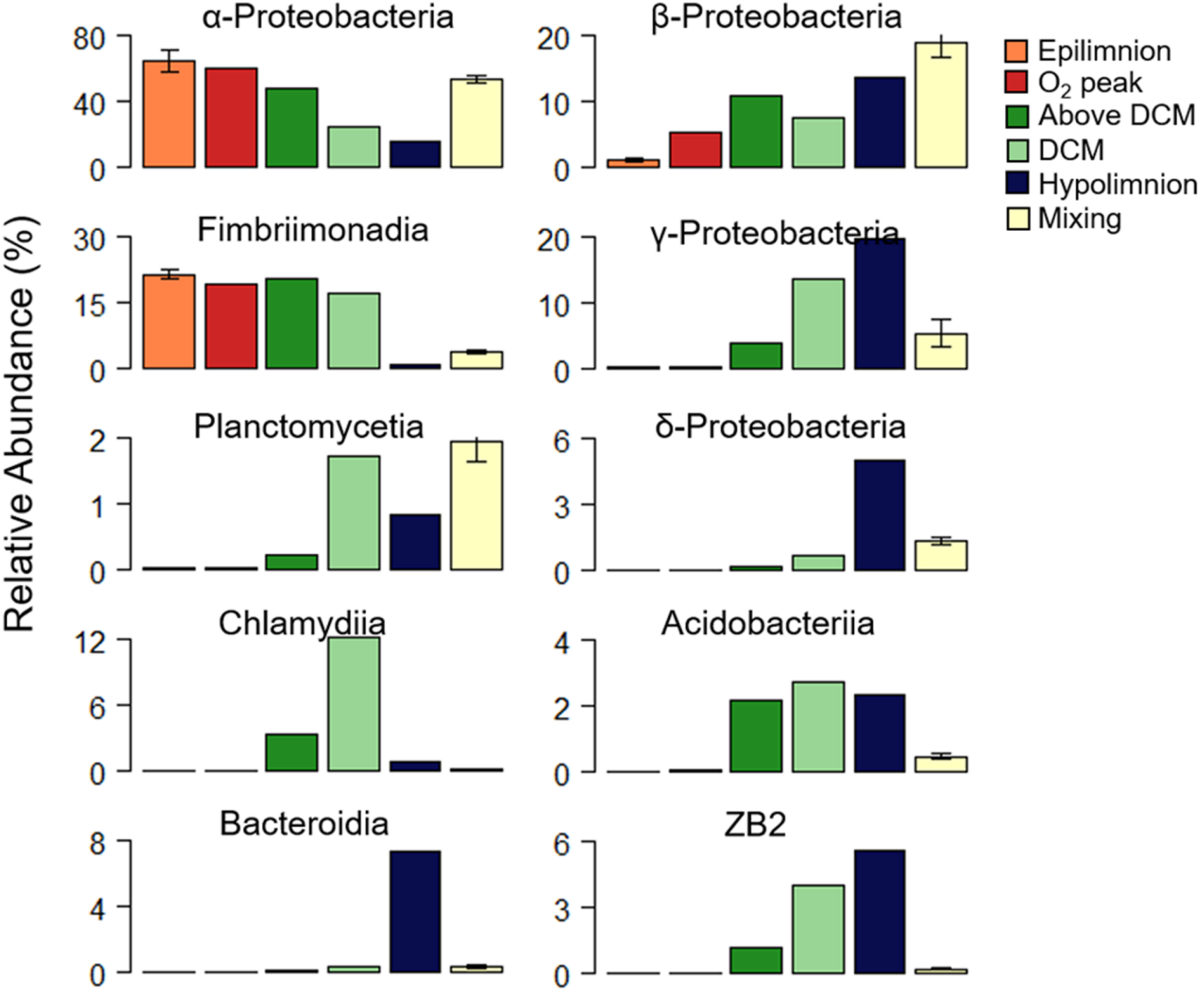
Relative abundance (%) of most abundant bacterial classes in the microbial niches distinguished in El Sancho during stratification (October 2013) (*n* = 7) and three samplings during mixing (January–February 2014) (*n* = 21).

Proteobacteria (47%–75% of the total community) dominated all microbial niches at the phylum level (Figure S4), being likely very important in shaping the functional response of El Sancho reservoir microbiota to the environmental changes with depth and between seasons. *Proteobacteria* classes differed clearly in their spatiotemporal niche occupation. *α‐Proteobacteria* dominated the upper layers and the mixing period and decreased with depth in both seasons, while *β‐*, *γ‐* and *δ‐Proteobacteria* increased with depth, especially during stratification (Figures 3 and S5). The versatility of *α‐Proteobacteria* regarding substrate demands and grazing resistance could explain their dominance in El Sancho microbiota during both seasons so it could be considered generalist (Percent et al., 2008; Yu et al., 2015).

Noteworthy is the high abundance of the poorly‐known phylum Armatimonadetes (~17% of the total bacterial abundance from the epilimnion to the DCM) and its class *Fimbriimonadia* in El Sancho. As far as we know, this class has never been reported before in acidic environments (Im et al., 2012). Other classes were more closely associated with specific niches, *Chlamydiia* with the DCM and *Bacteroidia* with the hypolimnion (Figures 3 and S5), suggesting narrower environmental requirements and potential specialist classes compared to others. Candidate divisions OD1 and its candidate class ZB2 are poorly studied groups, detected previously in the hypolimnion of a boreal lake and sulphur‐ and DOC‐rich anoxic environments (Newton et al., 2011; Peura et al., 2012). Surprisingly, Archaea represented a very small fraction of the OTUs in El Sancho reservoir and were pooled in the Others category (abundance <1%). This low abundance of Archaea contrasts with the abundances of around 5% of total OTUs found in typical AMD environments, with lower pH and higher metal concentration than in El Sancho (Kuang et al., 2013; Quatrini & Johnson, 2018).

During mixing, most bacterial classes reduced their relative abundance or almost disappeared, whereas others showed similar or even higher relative abundances (e.g., α‐, β‐*Proteobacteria, Planctomycetia*) (Figures 3 and S5). These changes highlight the dynamism of the microbial community at low spatiotemporal scales and the diversity of responses of the different taxa to the shifts in water column environmental variables with depth and season (Shade et al., 2012). This means that the strong biogeochemical differences between microbial niches in the water column select specific taxa from the reservoir microbiota even in the presence of high dispersal rates.

### Bacterioplankton abundance and single‐cell characteristics

**FIGURE 4:**
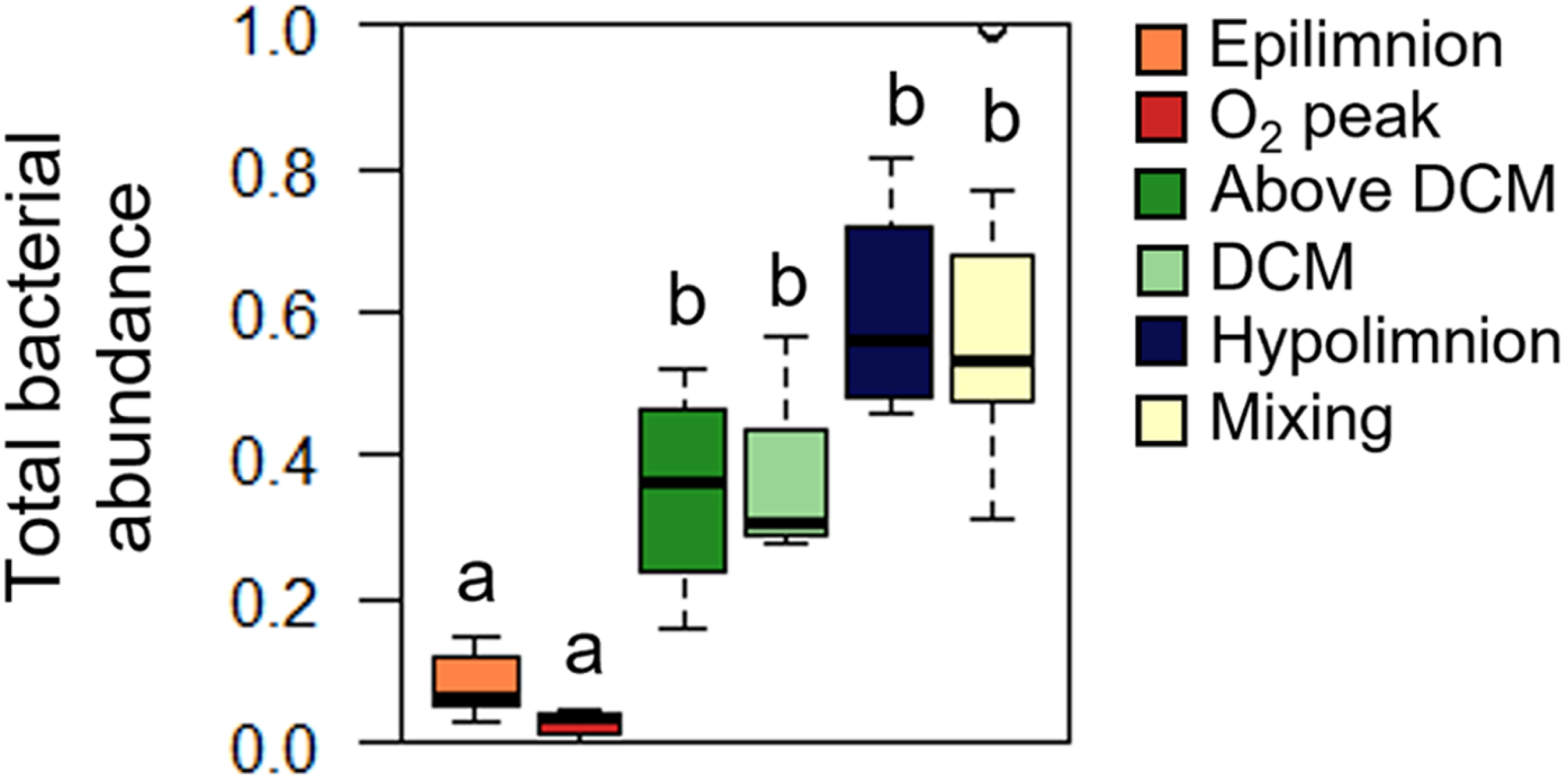
Box plots showing the normalized total bacterial abundance (0–1) in the microbial niches discriminated during stratification (September–October 2013) (*n* = 28) and during mixing (January–February 2014) (*n* = 21) in El Sancho reservoir. Lower to upper values are indicated, respectively, 10%, 25%, 50% (median), 75% and 90%. One‐way ANOVA indicated significant differences between niches (*F*_5,43_ = 24.37, *p* < 10^−5^). Tukey test post hoc homogenous groups (niches not significantly different at the *p* = 0.05 level) are shown with the same superscript letters.

Total bacterial abundance (HNA + LNA abundances) in El Sancho ranged between ~1 and ~6 × 10^8^ cells/L (Figure S6A,B). These bacterial abundances were within the range reported in other acid lakes and reservoirs, typically one or two orders of magnitude lower than those reported in neutral lakes (Kamjunke et al., 2004; Salcher et al., 2010). The vertical profile of total bacteria abundance showed clear seasonal differences; and an increase with depth during stratification, whereas no clear pattern was observed in mixing (Figure S6). Total bacterial abundance in the epilimnion and ‘O_2_ peak’ was significantly lower than in the other microbial niches as suggested by a one‐way ANOVA followed by post hoc comparison (Figure 4). The increase with depth in total bacterial abundance coincided with the patterns observed in bacterial diversity and richness at different taxonomic levels (Figures 3 and S3–S5) and is likely due to the higher organic substrate availability and diversity with depth which stimulate microbial activity and growth (Table S1, Soria‐Píriz et al., 2019) as observed previously in comparable environments (Kamjunke et al., 2004; Salcher et al., 2010).

**FIGURE 5:**
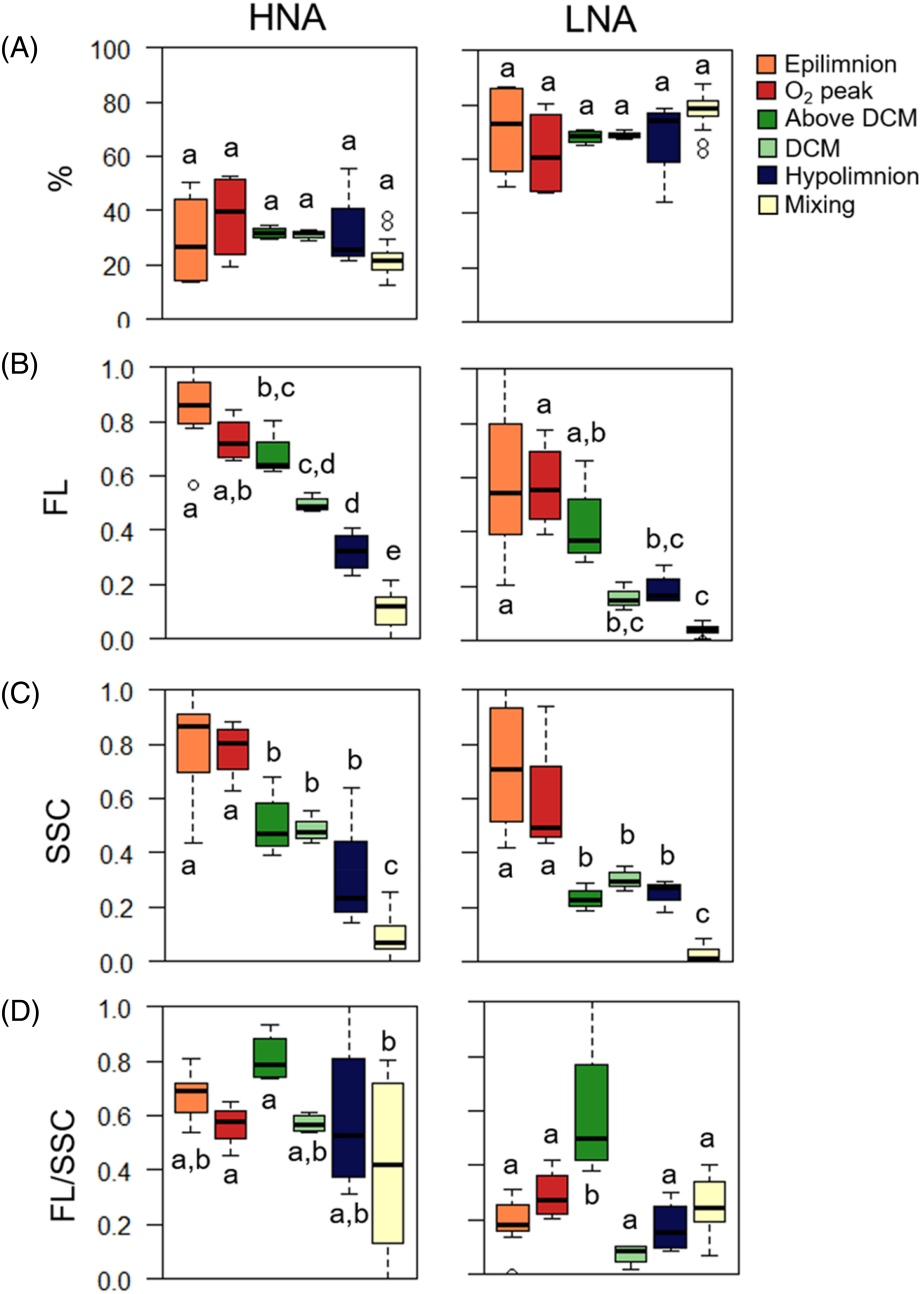
Box plots showing (A) the per cent respect total bacteria (%), (B) cell content of nucleic acid (FL), (C) cell side scatter (SSC) and (D) FL normalized by cell size (FL/SSC) of HNA and LNA cells both for the microbial niches during stratification (September–October 2013) (*n* = 28) and during mixing (January–February 2014) (*n* = 21) in El Sancho reservoir. All variables were normalized (0–1) except the % of LNA and HNA. Lower to upper values are indicated, respectively, 10%, 25%, 50% (median), 75% and 90%. One‐way ANOVA indicated significant differences between niches (*F*_5_,_43_ = 133.3–28, *p* < 10^−5^). Tukey test post hoc homogenous groups (niches not significantly different at the *p* = 0.05 level) are shown with the same superscript letters.

Since two bacterial subpopulations, HNA and LNA cells were distinguishable in the bacterioplankton community, further quantitative analyses were done considering this distinction. Surprisingly, the % HNA and LNA bacteria in El Sancho were rather constant, ~30% and ~70% respectively, both seasonally and with depth (Figure 5A), in contrast to what has been observed in other lakes (Nishimura et al., 2005; Salcher et al., 2010). Ordination analysis based on the abundance of HNA and LNA and their single‐cell characteristics (SSC and FL) distinguished samples between niches along CCA1 (74.6%, *F*_1,43_ = 154.6, *p* = 0.001) (Figure 2B). Forward selection identified temperature, DOC, pH and Fe, as the main environmental variables explaining together 74% of the total variation. Therefore, this clearly shows that the different microbial niches supported significantly different microbial communities regarding taxonomic composition, as discussed in the previous section, which differed in HNA and LNA abundances and their single‐cell characteristics as well (Figure 2).

A closer analysis of the changes in HNA and LNA abundances and single‐cell characteristics between the different microbial niches in El Sancho revealed interesting features and opened new ecological questions. Differences between niches were evident in the absolute abundances of the bacterioplankton and the single‐cell characteristics, that is, FL (a proxy of nucleic acid cellular content) and SSC (a proxy of size, granularity, and complexity of the cell) values of both HNA and LNA fractions, but not in the relative abundance of both fractions (Figures 4 and 5). SSC and FL of both HNA and LNA fractions decreased significantly with depth during stratification and reached the lowest values in mixing (Figure 5B‐C). This suggests a decrease in mean cell size (estimated by SSC) and nucleic acid content per cell (estimated by FL) with depth during stratification and from stratification to mixing. Since bacteria cell size within a given species is positively correlated with growth rate and nutrient availability (Vadia et al., 2017; Weart et al., 2007), the decrease in mean cell size from stratification to mixing was expected and consistent with the lower organic nutrient availability and lower temperature during mixing (Table S1, Soria‐Píriz et al., 2019). Despite the bacterial biovolume (μm^3^, data not shown) being similar to the ones found in other ecosystems (Ruiz‐González et al., 2013, Mariazzi et al., 1998, Šimek and Straškrabová, 1992), the mean decrease of cell size (SSC values as a proxy) and nucleic acid content with depth from the epilimnion was surprising and it is contrary to the typical pattern in neutral reservoirs and lakes (Salcher et al., 2010; Salvador Hernández‐Avilés et al., 2012). In addition, it seems counterintuitive given the increase of nutrients with depth (Table S1), which was expected to favour higher bacterial growth rates (Soria‐Píriz et al., 2019) by larger bacteria with more nucleic acid per cell (Vadia et al., 2017; Weart et al., 2007).

Several alternative explanations could support the unexpected inverse patterns observed in the changes in cell size and nucleic acid content with depth during stratification. The decrease in temperature with depth and taxonomic changes are unlikely to play a relevant role. The thermal gradient during stratification occurs in both neutral and acid lakes and reservoirs and, therefore, cannot be the cause of the decrease in cell size and nucleic acid content in El Sancho. A decrease in mean cell size and nucleic acid content due only to the changes in the taxonomic composition cannot be ruled out but seems unlikely given the fact that most taxa were present in several niches. The large bacterial cell size in the epilimnion might be due to a photoprotection mechanism against high levels of UV radiation due to the high transparency of the water column and summer irradiance levels in El Sancho (Soria‐Píriz et al., 2019). Bacteria cell size and complexity increase as a photoprotection mechanism against high UV radiation due to the accumulation of UV protective mycosporine‐like amino acids and because larger cells have lower surface‐to‐volume ratios and therefore receive a lower effective UV dose (Arai et al., 1992; Goldman & Travisano, 2011). In addition, differences in grazing pressure and type of grazers between the niches might also contribute to explaining the observed decrease in bacterial cell size (McKie‐Krisberg & Sanders, 2014; Mitra et al., 2014). Further work is needed to evaluate the relative role of these and other ecological factors on the differences in bacterial cell size between the microbial niches.

The normalization of nucleic acid content by cell size, estimated by the FL/SSC ratio allows us to detect those communities with a higher relative content of nucleic acid and, therefore, with a potentially higher growth rate, independently of their cellular size than other communities. FL/SSC for HNA and LNA differed little among the niches, except for the Above DCM layer where a peak in FL/SSC was observed for both bacterial fractions (Figures 5D and S7A–C). Our results suggest that FL/SSC could be a valid flow cytometry proxy for bacterial growth. FL/SSC was higher in the Above DCM, where we expected a higher heterotrophic growth rate, coinciding with the position of maximum phytoplanktonic growth rate and likely higher abundance of labile organic substrates (Soria‐Píriz et al., 2019). Similarly, in the ocean, higher bacterial activity occurs usually in the vicinity of DCMs (Scharek & Latasa, 2007; Van Wambeke et al., 2011).

### Relationship between bacterioplankton taxonomic composition, abundance and single‐cell characteristics across the microbial niches

The correspondence between the ordinations based on taxonomy and that based on the single‐cell characteristics of the bacterial community in El Sancho was high as shown by the Procrustes analysis (*r* = 0.55, *p <* 0.001) (Figure 2C). In fact, further PERMANOVA analysis showed that the most abundant classes corresponded to both SSC HNA and SSC LNA (Permanova_SSC HNA_: *R* ^2^ = 0.19, *p*‐value = 0.004 and Permanova_SSC LNA_: *R* ^2^ = 0.09, *p*‐value = 0.036) suggesting that size difference is related to taxonomic group. The congruence between changes in community taxonomic composition and single‐cell characteristics and abundance in space and time indicate a simultaneous response of the microbial community at multiple levels, from single‐cell to taxonomy (Comte & del Giorgio, 2009). The changes in the multilevel structure of the microbial community are likely the result of a complex interplay between bottom‐up and top‐down mechanisms, whose final result might be niche‐specific (e.g., the reasons behind the small cell size in the hypolimnion during stratification are likely different to those operating in mixing). Unfortunately, our data set does not include information on potential top‐down mechanisms, like interspecific competitions or grazing, which might affect bacterioplankton abundance and niche selection in El Sancho. Nonetheless, our results show that bottom‐up ecological factors, like changes in temperature, DOC, pH and Fe, not necessarily in the same order, explained the niche ordinations based on taxonomy and single‐cell characteristics (Figure 2A, B). These bottom‐up ecological factors have been shown to determine different features of bacterioplankton dynamics, like metabolic activity, growth rate, cell size, taxonomic composition and total abundance (del Giorgio & Gasol, 2008). The changes in these features likely contributed to the adaptation of the community structure from the same reservoir microbiota to the spatiotemporal variation in the microbial niches.

In addition to the selection (filtering) of species due to niche‐specific biotic and abiotic factors, differences in the dispersal rate have been shown to play an important role in the taxonomic structure of microbial communities (Lindström & Östman, 2011; Niño‐García et al., 2016a, 2016b; Stegen et al., 2012). However, here we showed that even with high dispersal rates in the water column, niche selection seems to be the main determinant of both the microbial community taxonomic composition and its single‐cell characteristics and abundances in El Sancho. These results at a low spatiotemporal scale and in the presence of high dispersal rates, as estimated from *K*_d_, contrast markedly with previous studies at larger scales. Finally, independently of the relative role of dispersal and niche filtering in the shape of microbial communities, the structure of the community changed at different organizational levels through space and time. Further work will be necessary to investigate: (a) the consequences of the changes in the structure of the community, at different structural levels, on the overall ecosystem functions played by microorganisms, and (b) the relative role that different ecological factors (i.e., biotic and abiotic niche dimensions) play in the regulation of the different structural levels of microbial communities.

## AUTHOR CONTRIBUTIONS

**Sara Soria Piriz:** Conceptualization; investigation; methodology; visualization; writing – review and editing; formal analysis; data curation; writing – original draft; validation. **Alfonso Corzo:** Writing – review and editing; writing – original draft; supervision; funding acquisition; project administration; resources; investigation; validation. **Juan Luís Jiménez‐Arias:** Formal analysis; writing – review and editing; methodology. **Juan M. Gonzalez:** Writing – review and editing; formal analysis. **Sokratis Papaspyrou:** Conceptualization; investigation; writing – review and editing; writing – original draft; validation; supervision.

## CONFLICT OF INTEREST STATEMENT

The authors declare no conflict of interest.

## Supporting information


**Data S1.** Supporting Information.

## Data Availability

Data used in this study are deposited in the Dryad Digital Repository as ‘Private for Peer Review’, which link is: https://datadryad.org/stash/share/9Qw79UgP9SJ2yY63UC9PCTC8aBX85JygDFglcRgUkwg. The data set includes three files .csv format. The first file includes the environmental data collection using a multiparameter probe (Temperature and Fluorescence) and an oxygen microsensor (Oxygen). The second file includes the environmental data collection using a Van Dorn acrylic bottle (Oxygen, carbon dioxide, ammonium, nitrate plus nitrite, chlorophyll a, particulate organic carbon and dissolved organic carbon) and pH (multiparameter probe). The third file includes the diversity index of the bacterioplankton community, the relative abundance of the more abundant phyla and classes and the abundance and single‐cell characteristics of the high and low nucleic acid content bacterial subgroups. Sequence data have been deposited in the NCBI database under the accession number PRJNA720891.
