# Supplementary material for: Niche selection in bacterioplankton: A study of taxonomic composition and single‐cell characteristics in an acidic reservoir
Source: Environ Microbiol Rep. 2024 Jun 28;16(4):e13255. doi: 10.1111/1758-2229.13255 (PMC11212339; doi:10.1111/1758-2229.13255)
Supplement: Supplementary file 1 — Data S1. Supporting Information. [file EMI4-16-e13255-s001.docx]

**Supplementary material**

**Tables**

**Table S1:** Mean ± standard error of temperature (T), dissolved oxygen (O_2_), pH, carbon dioxide (CO2), ammonium (NH4+), Nitrate + Nitrite (NOx), chlorophyll a (Chl *a*), particulate organic carbon (POC), dissolved organic carbon (DOC), potassium (K), magnesium (Mg) calcium (Ca), aluminum (Al), soluble ferrous iron (Fe^2+^, abbreviated as Fe), Zinc (Zn), copper (Cu), Manganese (Mn), Sodium (Na) and Silica (Si) and turbulent diffusion coefficient (K_d_) for six different microbial niches in El Sancho reservoir water column. Five of them are spatially separated along the water column during stratification (Sept-Oct 2013): epilimnion (0 - 10m depth), O_2_ peak (16m depth), above DCM (20m depth), DCM (22 - 24m depth) and hypolimnion (28 - 33m depth). The sixth one includes the whole water column during mixing (Jan-Feb 2014), representing a temporally separate environment from the rest. < LOD: below limit of detection. Data are from four samplings (n = 28) during stratification (Sept-Oct 2013) and from three samplings (n = 21) during mixing (Jan-Feb 2014) in El Sancho reservoir. Data used in each microbial niche are specified in the table.


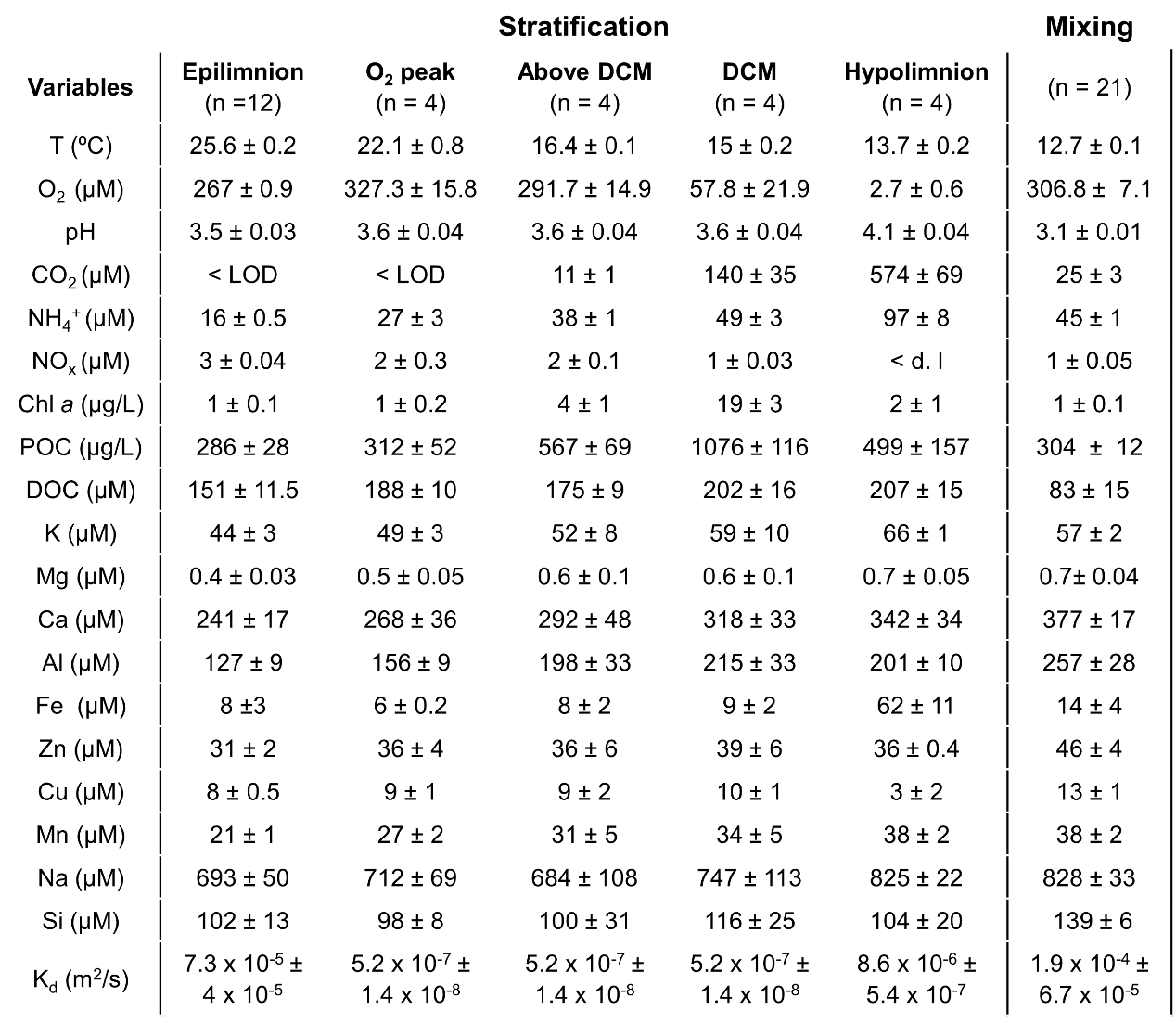


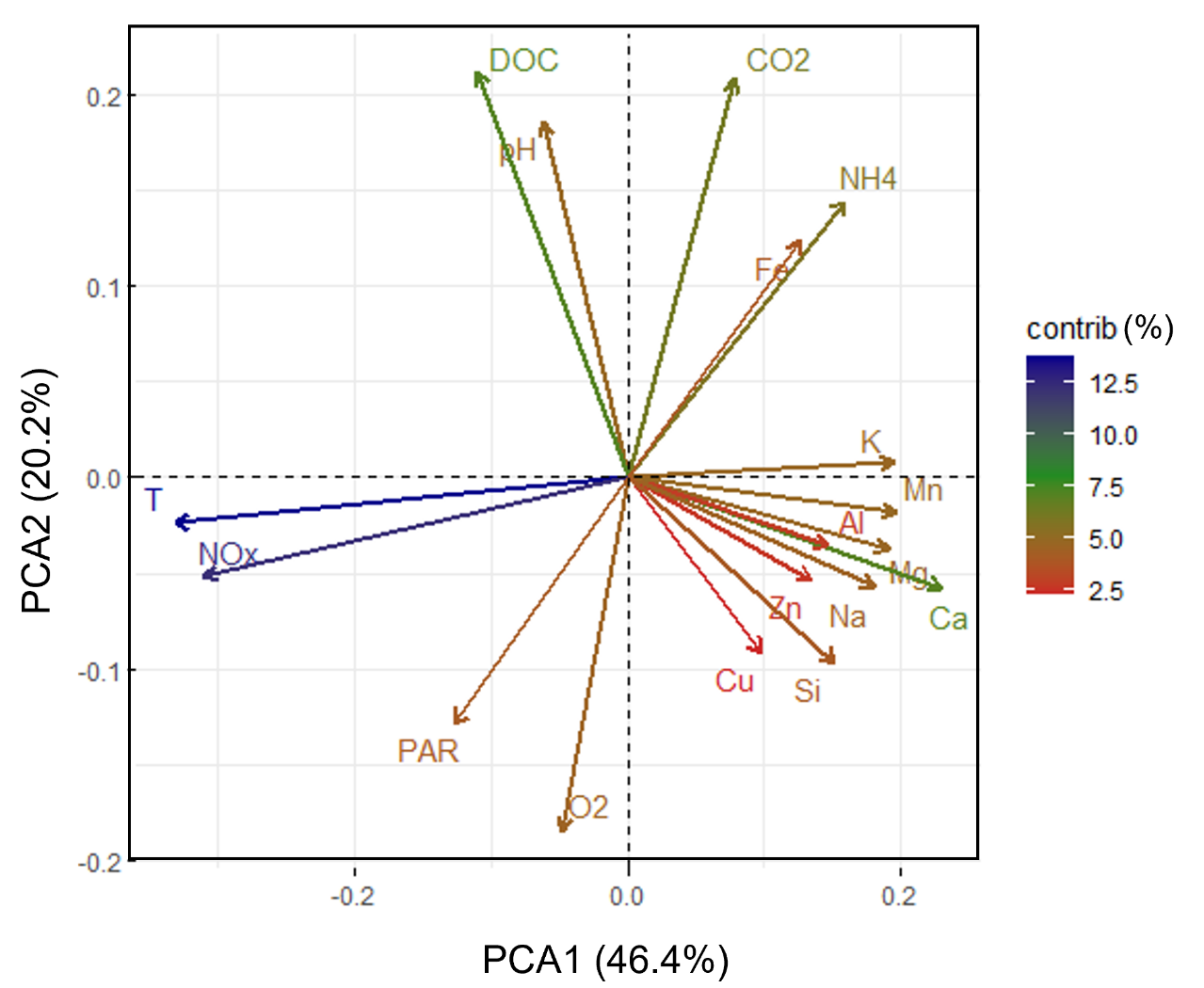


**Fig. S1:** Plot of the first two components of a principal component analysis (PCA) based on the Euclidean dissimilarity matrix of the abiotic variables to visualize their contribution (%) to the ordination. Abiotic variables: temperature (T), dissolved oxygen (O_2_), pH, carbon dioxide (CO2), ammonium (NH4+), Nitrate + Nitrite (NOx), dissolved organic carbon (DOC), potassium (K), magnesium (Mg,) calcium (Ca), aluminum (Al), soluble ferrous iron (Fe^2+^, abbreviated as Fe), zinc (Zn), copper (Cu), manganese (Mn), sodium (Na) and silicate (Si). Only variables that contributed > 10% in the first two PCA components were selected to identify potential niches. Data are from four sampling during stratification (September-October 2013) (n = 28) and three samplings during mixing (January-February 2014) (n = 21).


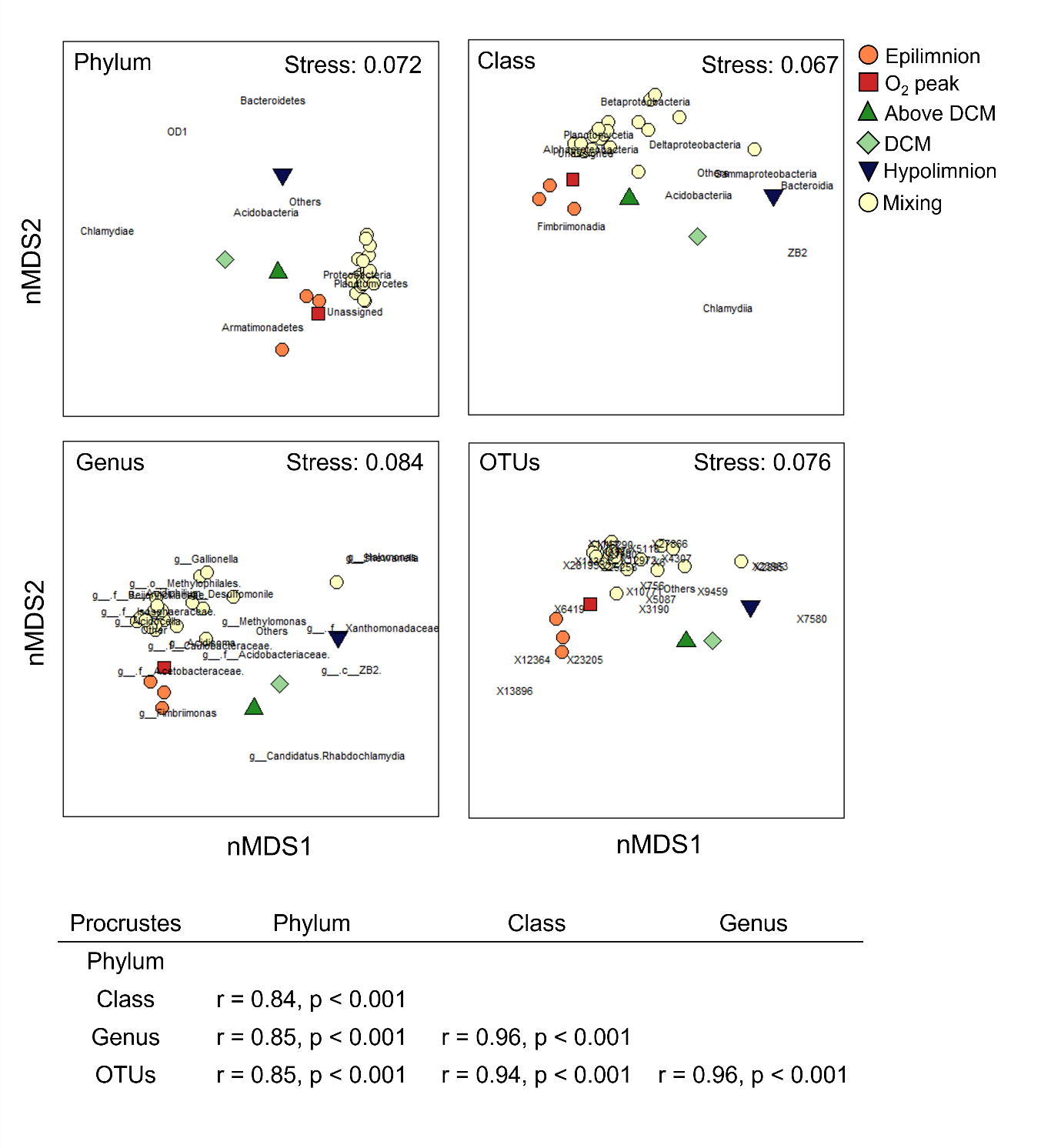


**Fig. S2:** Non-metric Multidimensional Scaling (nMDS) ordination analysis based on the Bray Curtis distance at different taxonomic levels (Phylum Class, Genus and OTU) of 16S rDNA. Data are from one sampling during stratification (October 2013) (n = 7) and three samplings during mixing (January-February 2014) (n = 21).


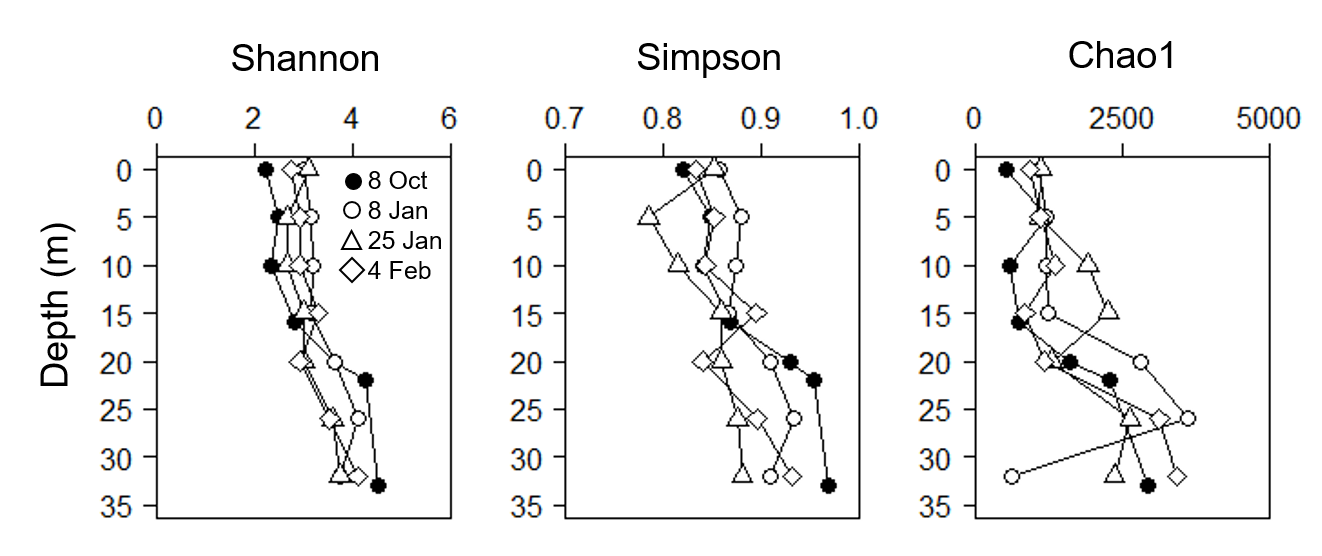


**Fig. S3:** Depth profiles showing the vertical distribution of the Shannon, Simpson, and Chao1 diversity indices calculated from 16S rDNA OTUs. Data are from one sampling during stratification (October 2013) (n = 7) and three samplings during mixing (January-February 2014) (n = 21).


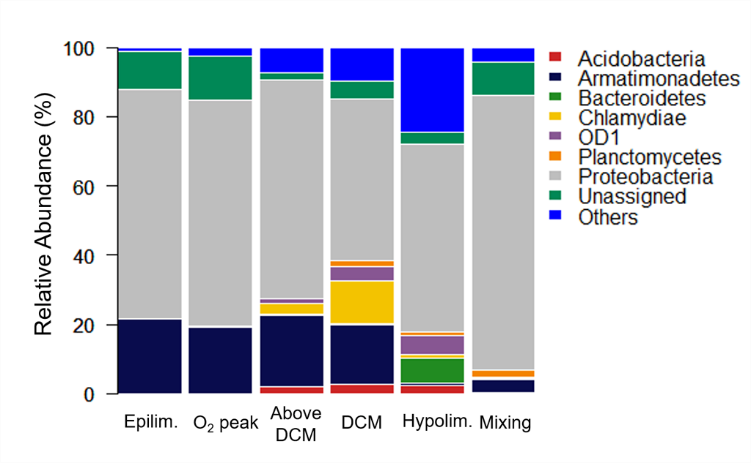
**Fig. S4:** Relative abundance of the most abundant bacterial phyla in the microbial niches in El Sancho reservoir during stratification and mixing. Bacterial phyla with an average relative abundance > 1% are shown, while “Others” includes all phyla with relative abundance < 1%. “Unassigned” included the unclassified bacterial phyla. Data are from one sampling during stratification (October 2013) (n = 7) and three samplings during mixing (January-February 2014) (n = 21).

**
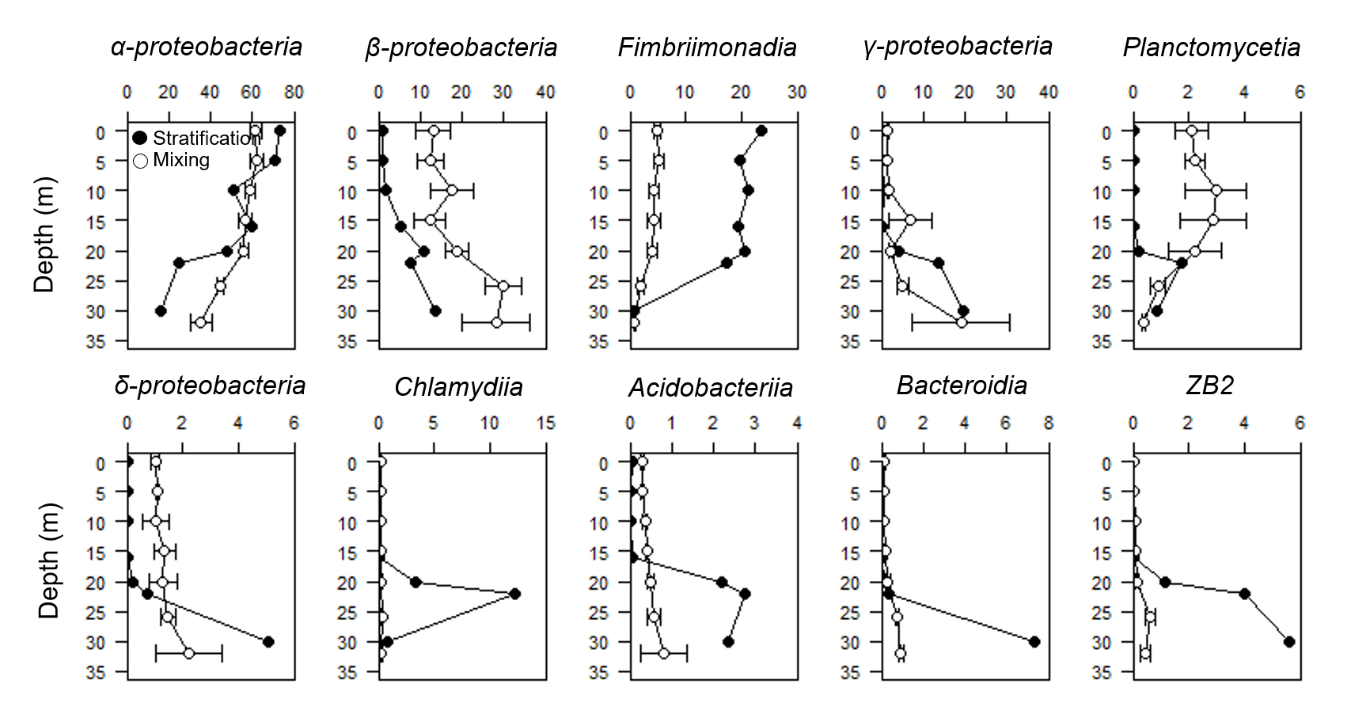
**

**Fig. S5:** Depth profiles showing the vertical distribution of the relative abundance (%) of most abundant bacterial classes determined by DNA-based techniques in El Sancho reservoir. Data are from one sampling during stratification (October 2013) (n = 7) and three samplings during mixing (January-February 2014) (n = 21).


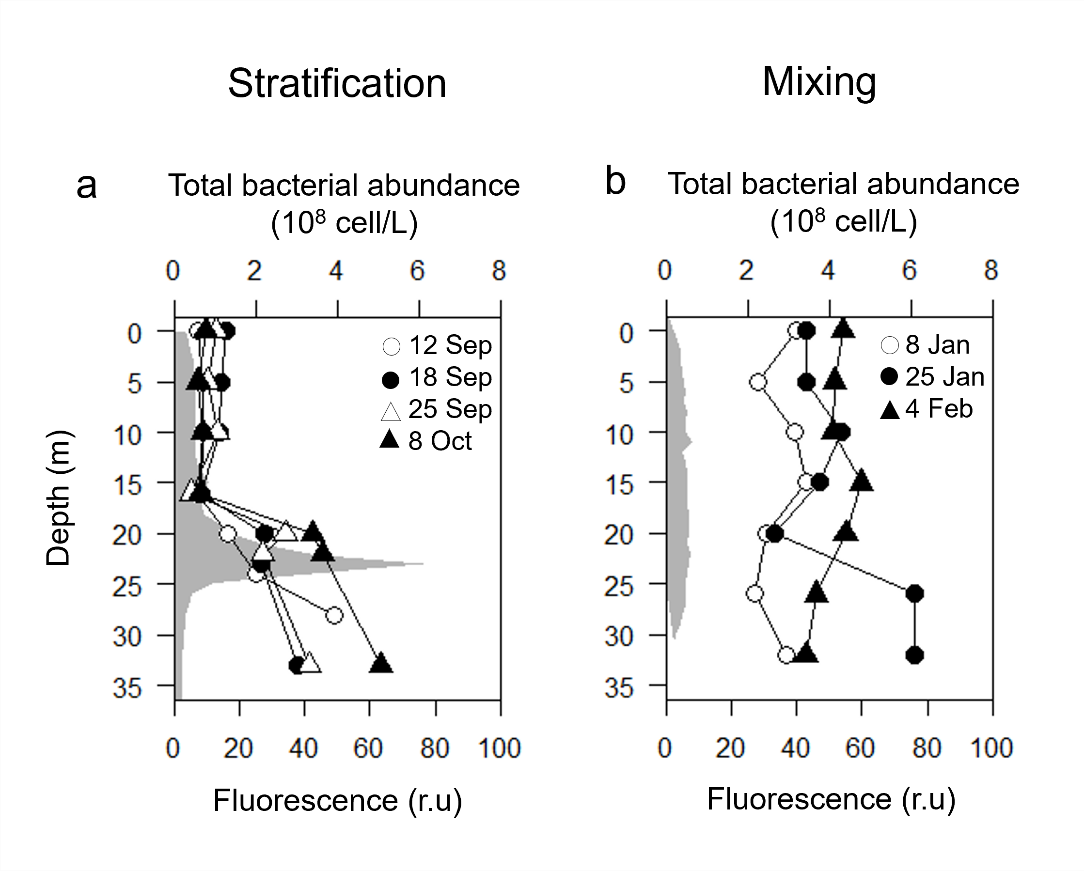


**Fig. S6**: Depth profiles showing the vertical distribution of total bacteria abundance (cell/L) in El Sancho reservoir during (a) stratification and (b) mixing. Fluorescence profiles (grey area) are also shown for spatial reference. Data are from four sampling during stratification (September-October 2013) (n = 28) and three samplings during mixing (January-February 2014) (n = 21).


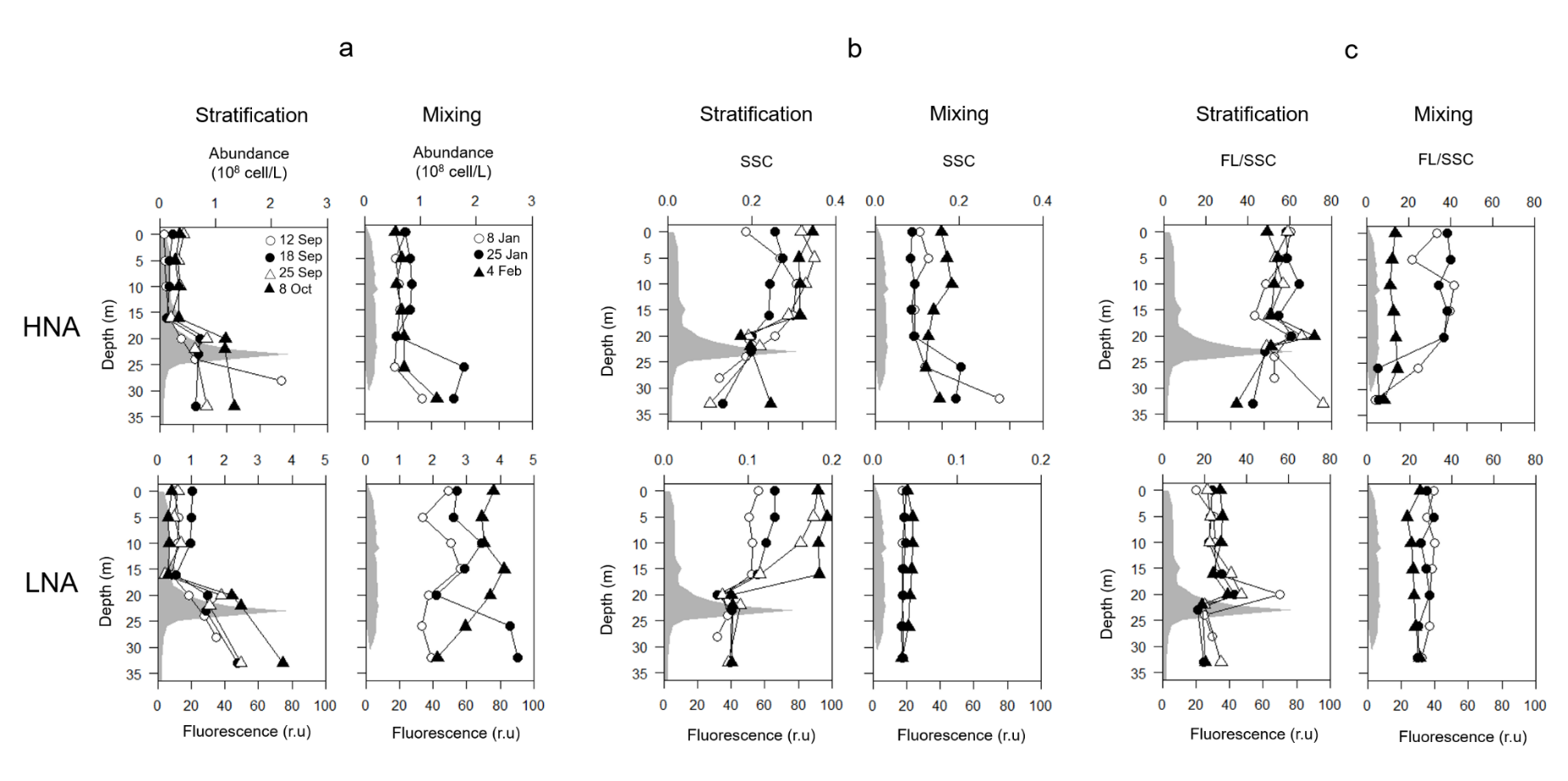


**Fig. S7:** Depth profiles showing the vertical distribution of (a) abundance, (b) SSC and (c) FL/SSC of HNA and LNA fractions in El Sancho reservoir Fluorescence profiles (grey area) are also shown for spatial reference. The abundance and single-cell characteristics of both HNA and LNA subgroups were determined by flow cytometry. Data are from four sampling during stratification (September-October 2013) (n = 28) and three samplings during mixing (January-February 2014) (n = 21).
